# Management of multiple sclerosis in older adults: review of current evidence and future perspectives

**DOI:** 10.1007/s00415-024-12384-3

**Published:** 2024-04-30

**Authors:** Kimberly A. DiMauro, Carol Swetlik, Jeffrey A. Cohen

**Affiliations:** Mellen Center for MS Treatment and Research, Cleveland Clinic, Neurological Institute, Cleveland, OH USA

**Keywords:** Multiple sclerosis, Aging, Late-onset multiple sclerosis (LOMS), Older adults with multiple sclerosis (OAMS), Disease-modifying treatments

## Abstract

**Importance:**

The prevalence of multiple sclerosis (MS) and aging MS patients is increasing worldwide. There is a need to better understand this MS sub-population, which historically is underrepresented in the literature. This narrative review examines the evolving demographics, disease course, and treatments for older adults with MS (OAMS) to address current knowledge gaps and highlight areas critical for future research.

**Observations:**

OAMS populations require special consideration by clinicians. Older individuals have different care needs than individuals with adult onset MS who are mid-life or younger. Comorbidities, an aging immune system, increasing neurodegeneration, decreasing neurologic reserve, changing benefit/risk relationship for disease modifying therapies (DMTs), and wellness require special attention to provide holistic comprehensive care. Active areas of research include potential cessation of DMTs and novel disease targets.

**Conclusions and relevance:**

This review highlights both the current knowledge and information gaps in the literature that are critical to understanding and properly managing OAMS. The aims are to inform MS clinicians in their current practice, as well as inspire future studies which are critical to providing quality and evidence-based care for OAMS.

## Introduction

Multiple sclerosis (MS) is a chronic central nervous system (CNS) autoimmune disorder with clinical onset typically in young adults ages 20–40. As the world’s population ages and survival increases, there is an increasing number of older adults with MS (OAMS). It is estimated that 2.8 million people are living with MS worldwide, with peak prevalence in the United States (US) ages 55–64 years, followed by ages 65–74 [1, 2]. In the US, it is estimated that approximately 10% of people with MS are over age 65, with that proportion likely to grow [3]. This increasing MS prevalence and recognition in less common populations has seen rise to various MS definitions, including adult onset MS for patients diagnosed between ages 18 and 50, late-onset MS (LOMS), defined as MS diagnosis after age 50, and very late-onset MS (VLOMS) defined as MS diagnosis after age 60 [4].

In addition to these more recent trends, our understanding of MS pathophysiology continues to evolve. Over time, the biology that drives relapses and magnetic resonance imaging (MRI) lesion activity, focal inflammatory demyelination, tends to become less prominent, while the biology that drives progression, diffuse nonresolving compartmentalized inflammation and neurodegeneration, becomes more apparent [5]. This change has implications for MS treatment, particularly in the older MS populations, as the currently available disease modifying treatments (DMTs) are less efficacious with the shift in pathophysiology [6].

Medical comorbidities can further complicate making the correct disease diagnosis in LOMS, as well as influence disease course and treatment. These elements add additional layers of complexity in the OAMS population for the clinician to consider. In this review, we discuss the current evidence regarding demographics, disease course, treatment management, and special considerations for OAMS in comparison to their adult onset counterparts. PubMed was searched using the terms: ‘multiple sclerosis, MS, late-onset, older adults with MS’ with no time or language limitations. The authors independently selected and assessed the eligible articles based on this criteria. References within these articles were additionally evaluated and included as deemed relevant.

## Epidemiology of MS in the older age group

The rising prevalence of MS has been noted worldwide, including individually in Canada, New Zealand, and US, among other regions [1, 7–9]. One large population cohort study in Manitoba demonstrated rising prevalence with relatively stable incidence of MS, which was interpreted to indicate decreased mortality in an aging population [7]. This is further supported by the observation that in recent decades MS patients’ life expectancy has increased, but still reduced compared to the general population, with suggested average reductions in lifespan of 6–10 years for patients with MS [10]. In other studies, the prevalence of MS patients age 65 and above has been estimated to be 10% in US and 18% in Italy [3, 11]. There is evidence of increasing diagnosis of MS over the age 60, with LOMS estimated to represent approximately 5% of cases [4, 12–14]. The reasons for these epidemiologic changes are likely multifactorial, including changes in global life expectancy, increasing access to health care, improved disease awareness and diagnosis, and evolving diagnostic criteria.

## Age-related immunosenescence in MS

Immunosenescence, which has been defined as a “multifactorial and dynamic phenomenon that affects both natural and acquired immunity,” is part of the aging phenotype [15]. While a full characterization of molecular underpinnings is beyond the scope of this review, immunosenescence arises when an expansion of abnormal immune cell populations, both innate and adaptive, leads to impaired immune system function and resultant increased risk of infection and cancer [15]. Simultaneously, low-grade, sterile chronic inflammation, termed "inflammaging," emerges [16]. A reduced ability to generate effective immune responses against infections and vaccinations results, despite increased levels of inflammatory cytokines and activity of the innate immune system [15].

People with MS have premature immunosenescence and reduced immune function with accompanying premature aging markers, including telomere length reduction, iron accumulation, and increased oxidative stress [10, 17–19]. These markers are associated temporally with neurologic disability progression and are thought to contribute to gradual accrual of disease burden despite decreased frequency of relapses in later life [10]. Recent publications highlight epigenetic age acceleration, specifically in B-cells in people with MS [20]. Senolytics and regenerative strategies targeting these mechanisms may provide novel treatment opportunities [21, 22].

## Disease behavior in older adults with MS

Historically, a progressive MS phenotype has been considered the most common presentation of patients with LOMS. However, recent studies highlight that a relapsing course is not uncommon. In one retrospective cohort analysis of 2261 patients with LOMS, primary progressive MS (PPMS) represented 26.7% of all cases, but relapsing remitting MS (RRMS) was still most common phenotype overall (66.4%) [23]. LOMS can continue to exhibit active focal inflammatory lesion activity despite a later onset [24]. CSF profiles, however, have shown less pleocytosis (34% in LOMS compared to 67% in adult onset MS) [25]. These factors should be considered when evaluating an older patient with suspicion of MS, as ultimately the spectrum and phenotype of MS disease in the older population can vary.

Brain volume loss also appears more significant in OAMS and LOMS. Brain tissue damage with parameters of brain parenchyma fraction and gray matter fraction were more advanced in LOMS when compared to an adult onset MS group [26]. This study also found lack of correlation between age and brain atrophy parameters and excluded patients with known vascular comorbidities. Both of these observations suggest that there are significant differences in brain atrophy between adult onset MS and LOMS patients, not solely due to age or comorbid microvascular ischemic disease. Aging MS patients accrue more diffuse brain atrophy, which can be manifested as ventricular expansion [27, 28]. Interestingly, a recent study examining apathy and its relation to caudate volume in OAMS versus controls found that lower caudate volumes were significantly associated with increased apathy in the OAMS group [29].

In terms of disease outcomes, population-based cohort studies have shown an association of later MS diagnosis with greater risk of disability progression [23, 30]. Disease course in LOMS has also shown faster progression to disability [13]. LOMS has been shown to have increased risk of reaching higher Expanded Disability Status Scale (EDSS) milestones. Thus, a better understanding disease course and management in the older age group is paramount [29].

## Diagnosis

MS diagnosis in older populations can utilize the 2017 McDonald criteria, as in the more typical age range. Careful consideration of potential misdiagnosis, however, is of particular importance in the older population given some of the features that can differ in this group. Due to increased prevalence of comorbid conditions in OAMS, vascular risk factors (such as hypertension, hyperlipidemia, diabetes mellitus) leading to stroke and/or microvascular ischemic disease should be strongly considered in the differential of older patients with new neurologic symptoms or abnormal neuroimaging [31]. Spinal spondylosis (contributing to myelopathy), nutritional deficiencies (leading to a variety of manifestations), ophthalmic conditions (producing visual disturbances), and degenerative disorders (contributing to gait impairment or cognitive manifestations) are additional conditions that should be paid particular attention throughout evaluation.

**Fig. 1:**
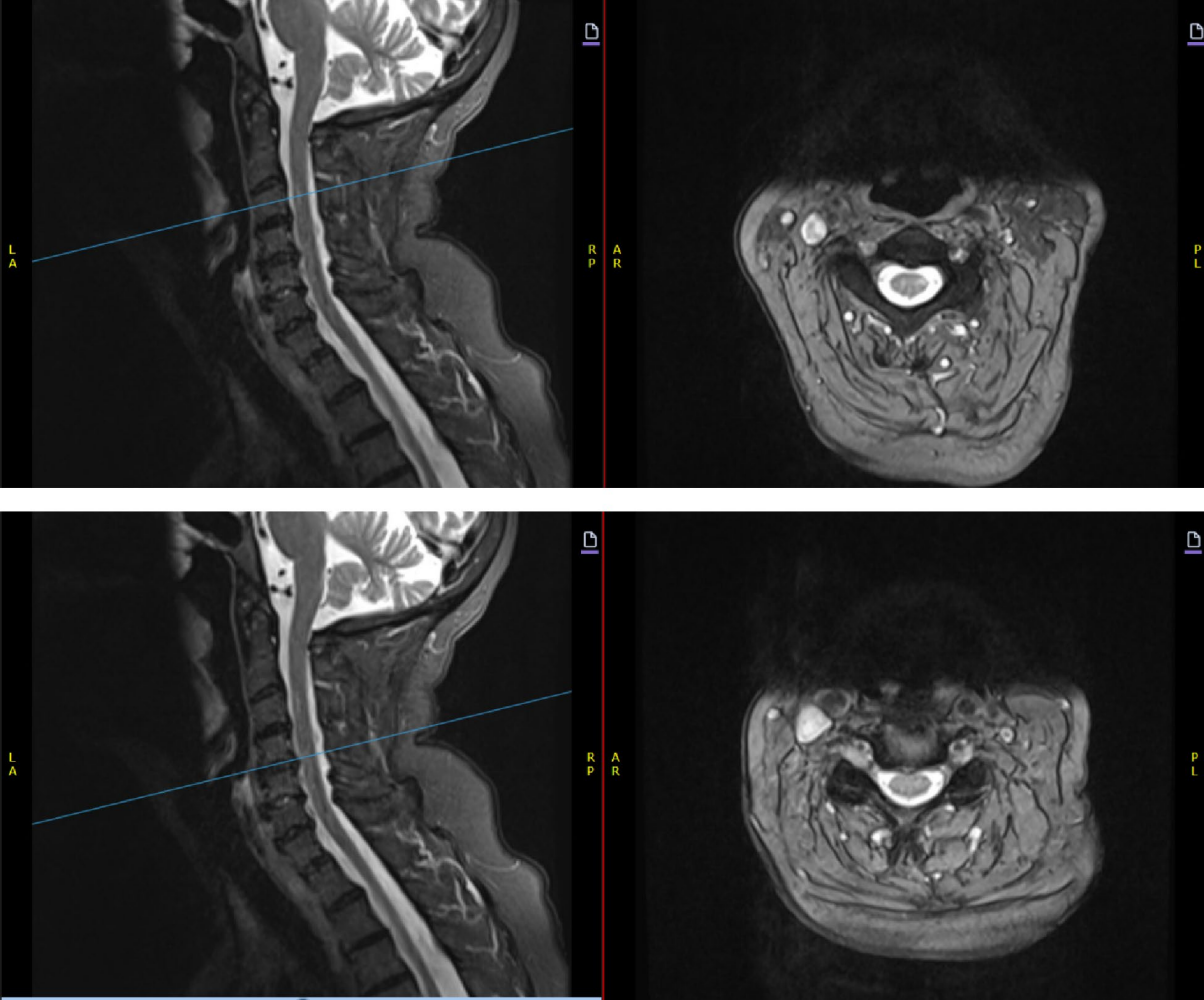
Degenerative disc disease changes co-occur with demyelinating lesions in patients with MS. Note demyelinating-related cord signal changes can arise at levels associated and not associated with structural spinal cord disease

In older MS populations, there is an association between accrual of spinal cord lesions with increasing disability progression [25, 32, 33]. Prevalence of myelopathy is higher in older populations in general, with 9.1% occurring in patients > 70 years of age, compared with 0.6% in patients < 20 years; additionally, recent studies confirm degenerative spine changes are present in most individuals after the 6th decade of life [34]. Spinal stenosis also may impact cord appearance, and a suggested association between segments of the spinal cord with moderate cervical stenosis and segments with MS lesions has been raised, as spinal segments with at least grade 2 stenosis  were significantly associated with presence of an MS lesion in the same segment [35]. Careful attention must also be given to spinal cord imaging, as spondylotic myelopathy is one of the most common MS mimics in the aging patient (Fig. 1). Tumor and amyotrophic lateral sclerosis also warrant consideration [36]. An older age should not preclude consideration of a LOMS-related myelopathy if history and imaging are consistent, and conversely a myelopathic picture in an OAMS should not prevent consideration of other causes.

Regarding brain imaging characteristics in the OAMS and LOMS populations, one should expect multifocal supratentorial and infratentorial lesions with similar demyelinating features as seen in other MS populations [25]. The clinician needs to be cognizant of chronic microvascular white matter changes, however, which increase in the older populations, particularly those with vascular risk factors. In a cohort of 338 MS patients, 41 (12%) were misdiagnosed, with cerebrovascular disease being the most common alternate diagnosis [37]. In this study, older age was found to be more common in misdiagnosed patients (odds ratio 1.2, 1.06–1.35, *p* = 0.003).

## Disease therapy in older adults with MS

Most of the clinical trial data serving as the basis for regulatory approval of MS DMTs were obtained in patients younger than age 55 years, limiting generalizability beyond the 6th decade of life [10]. Many trials excluded patients > 45 years or > 50 years [38–44]. Limited observational data are available for some medications in both OAMS and LOMS. Ultimately, formal prospective studies are needed to ascertain risk/benefit of DMT use in OAMS accurately [45, 46]. Aging also can affect pharmacokinetics and pharmacodynamics of drugs, and, hence, inclusion of OAMS in formal studies of these pharmacologic parameters also are needed in the older population [47].

In addition to inadequate representation in clinical trials, OAMS often experience a progressive MS course, for which few treatment options are available. Targets for neurodegeneration and diffuse nonresolving inflammation that may drive progression are currently under research. The only regulatory-approved option for PPMS currently is ocrelizumab [48]. A number of other agents have been evaluated in PPMS and secondary progressive MS (SPMS), including interferons, glatiramer acetate, mitoxantrone, cladribine, fingolimod, and rituximab. Recommendations specifically for OAMS, whether progressive disease has overlaying inflammatory activity or not, are lacking [49, 50].

Therefore, use of currently available DMTs in OAMS requires balance of likelihood of benefit against risk, such as infection including progressive multifocal leukoencephalopathy (PML). The rate of seroconversion to John Cunningham virus is 10.8% per year and, therefore, risk increases with age [51]. In patients who were new to treatment with natalizumab, age was found to be a risk factor for natalizumab-related PML, as well as prior immunosuppressant use. Those over 50 years of age had a double in hazard ratio for earlier PML onset [52]. This elevated PML risk also might apply to OAMS treated with fingolimod [53].

### Role of comorbidities to guide treatment selection

Comorbidities play an important role in MS treatment selection and, despite the paucity of data from RCTs, may be particularly critical when deciding on DMT in OAMS (Table 1) [54 –64]. As discussed below, there is increased risk of disability progression in patients with multiple comorbidities. Simultaneously, multi-drug regimens and DMT side effects could negatively impact this population, creating a paradoxical increased need for DMT given a more aggressive disease course and potential increased risk of deleterious side effects in patients with comorbidities. In one retrospective observational analysis, there was decreased likelihood of DMT initiation in patients with anxiety or ischemic heart disease [65]. There also was decreased likelihood of initiating a DMT as the number of comorbidities increased. Interestingly, there were opposite trends for patients with depression, as this subgroup was found to be 13% more likely to initiate DMT. While comorbidities are not unique to older patients, and a full review of comorbidities and interaction with all DMTs used for MS is beyond the scope of this review, we highlight several key circumstances to consider.

**Table 1:** Comorbidities often seen in older patients that deserve consideration when selecting disease modifying therapy in patients with MS. DMT = disease modifying therapy

DMT	Comorbidities that need to be considered
Interferon beta	Spasticity, depression, suicidality, seizures, headaches, congestive heart failure, pulmonary hypertension [54]
Glatiramer acetate	No major impact on pre-existing comorbidities known [55]
Teriflunomide	Hypertension, peripheral neuropathy, renal failure, hyperkalemia, contraindication in severe hepatic impairment, pregnancy [56]
Sphingosine 1-phosphate receptor modulators	Hypertension, headaches, macular edema, bradyarrhythmia, restrictive lung disease, contraindication in severe untreated obstructive sleep apnea, second/third degree atrioventricular block (without a pacemaker, and if myocardial infarction, unstable angina, stroke, transient ischemic attack, or decompensated heart failure has occurred in past 6 months), concurrent use with monoamine oxidase inhibitor in ozanimod, CYP2C9*3/*3 genotype in siponimod [57–59]
Fumarates	Dyspepsia, irritable bowel syndrome, inflammatory bowel disease, preexisting liver injury [60]
B-cell depleting therapies	Colitis, hypogammaglobulinemia, contraindication in active hepatitis B infection [61]
Natalizumab	No major impact on pre-existing comorbidities known, contraindication if history of progressive multifocal leukoencephalopathy [62]
Cladribine	No major impact on pre-existing comorbidities known, contraindication in chronic infections such as HIV, hepatitis, tuberculosis, pregnancy, active malignancy, and patients still of reproductive potential who do not plan to use effective contraception [63]
Alemtuzumab	Elevated liver function tests, contraindication in HIV or other active infection [64]

Vascular comorbidities, given their frequency, should be considered prior to DMT initiation. Sphingosine 1-phosphate receptor (S1PR) modulators are associated with cardiovascular adverse events (bradyarrhythmia and atrioventricular conduction slowing) with initiation of treatment and secondary hypertension, both of which may be of increased concern in OAMS [66]. Similarly, abnormal liver function tests, neutropenia, and hypertension were also reported as adverse events with teriflunomide in phase II and III studies [67].

Risk for infection should also be considered on a case-by-case basis and certain medications may increase risk more than others. Natalizumab, along with fingolimod and dimethyl fumarate, was associated with a 59% increased risk of infection related physician claims in a population-based study [68]. When assessed individually in this same study, natalizumab was the only DMT for which the increased infection risk was significant. Alemtuzumab carried higher risk of infection, malignancy, and mortality for patients over 45 years of age [69]. Cladribine has not been shown to be associated with increased risk of adverse events, including lymphopenia, in patients over 50 years compared with younger patients [70]. Intriguingly, when using ocrelizumab, age was not predictive of IgG levels below the lower limit of normal [71]. In an additional retrospective cohort study of 185 MS patients on ocrelizumab, older age was associated with reduced infection risk; 16% of these patients were > 55 years (mean age 43 years, SD 10.6). Younger age, longer MS disease duration, and increased EDSS score were associated with increased odds of antimicrobial use, with younger patients particularly more prone to upper respiratory infections [72].

### Discontinuation of therapy

There is uncertainty regarding if and when to discontinue DMTs in OAMS. There is increased risk with DMTs with older age, but the tradeoff of risk versus benefit is not well established in OAMS, particularly with stable disease. Multiple cohort studies have addressed DMT discontinuation in OAMS. In one study, 216 patients were analyzed who discontinued DMT after treatment for at least 6 months, including 81 patients (37.5%) > 55 years old [73]. In the total cohort analysis, 32.9% patients with prior stable disease demonstrated new accumulation of disability after stopping DMT. This study, however, lacked a control group who were continued DMT, and OAMS represented a minority of studied population. In a population-based MS registry study, 132 “stoppers” of DMT for > 3 months and 366 “stayers” with DMT discontinuation < 3 months were analyzed with a median follow-up 6 years [74]. All patients analyzed were > 50 years of age. After propensity matching, there was no difference in risk of relapse or increase in EDSS between the two groups, although progression to EDSS 6.0 was 3.3 times greater in the “stopper” group (adjusted hazard ratio 3.29, 2.22–4.86, *p* < 0.0001).

DISCOMS was a multicenter, randomized, non-inferiority trial which sought to determine whether risk of disease activity is increased in MS patients age 55 years or older with no recent disease activity (defined as no MS relapse within 5 years or new MRI lesion within 3 years) who discontinue DMT compared to those who remain on DMT [75]. Participants who discontinued DMT were found to have a 7% higher rate of disease activity, either with new lesions on MRI or a relapse within 2 years, compared to those who continued DMT. There was no difference, however, in disability progression (11% in DMT continuation arm versus 12% in DMT discontinuation arm).

Other trials exploring DMT discontinuation are ongoing. While not specific to the OAMS population, DOT-MS (NCT04260711) is a clinical trial investigating discontinuation of DMTs in participants 18 years or older, with clinical and radiographic stability for 5 years. Preliminary data including 89 participants with average age in their early fifties showed that in the discontinuation arm 17.8% had notable new disease activity, 7 of which had signs of substantial MRI activity, defined as three or more new total lesions or two or more new lesions with active inflammation [76]. These findings ultimately lead to trial termination in March 2024, and participants will continue to be followed in an observational matter after restarting their previous DMT. STOP-I-SEP (NCT03653273) evaluates discontinuation of DMT in participants with SPMS age 50 or older and clinical and radiographic stability for 3 or more years.

With the above knowledge in hand, for MS patients who are 55 years of age or older and have been stable for 3–5 or more years on therapy, it is reasonable to discuss discontinuation of DMT, with counseling of associated risks. Despite lack of increased risk of relapse in cohort studies of participants over 60 years, in clinical practice some patients may prefer to remain on DMT. In our experience, the decision of DMT discontinuation must be made on an individualized basis.

### Symptomatic treatment

In addition to decisions about DMT, there also is need for concomitant symptomatic management in many patients. There is currently limited research evaluating the benefits, risks, tolerability, and effects on quality of life of commonly used symptomatic treatments in the aging MS population. Despite this knowledge gap, symptomatic treatments remain an important part of management in OAMS. Careful risk/benefit evaluations must be applied when using oral medications, to which risk of drug–drug interactions and side effects may be elevated in the aging population depending on the agent. For instance, use of anticholinergics for detrusor hyperactivity from MS can exacerbate cognitive impairment, which may be of particular detriment in older patient populations given increased likelihood of dementias and MS-related cognitive changes [77]. In addition to medications, physical and occupational therapy are important components of MS care, particularly in older age groups who suffer from multifactorial gait instability or impairment of hand function. Awareness of patient comorbid conditions, for example osteoarthritis or heart failure, may limit some of patient’s capabilities in therapy from an MS standpoint.

## Special considerations: comorbidities and the aging process in patients with MS

Comorbidities are relevant to both diagnosis and disease management in older patients. They are common; a study of patients with MS showed 44% of patients had comorbidities (cardiovascular, respiratory, musculoskeletal, diabetes, and others) [78]. Simultaneously, comorbidities may cloud the diagnostic picture. They may lead to delay in diagnosis, particularly in patients with LOMS, if MS plaques are mistaken for age- and comorbidity-related white matter hyperintensities on MRI as a result of microvascular disease, migraine, or if attributed to other CNS diseases when presenting with a neurologic complaint. Studies to validate the 2017 McDonald criteria were performed largely in patients under 50 years of age and, therefore, clinical and imaging manifestations related to comorbidities over the age of 50 were less represented [79]. Diagnostic delay has been shown to increase if obesity, smoking, or physical (vascular, autoimmune, musculoskeletal, gastrointestinal, visual) or mental comorbidities were present, even after stratifying by age of symptom onset (with the oldest category > 40 years) [80].

Further research on the interaction of comorbidities with MS over the lifespan is needed. Systematic review of comorbidities in patients with MS confirms many studies do not include the age distribution of the study population [81]. Other studies on comorbidities include age as a covariate when evaluating the impact of comorbidity on outcome, but do not report its significance or interactions. In general, studies of the impact of early and late-onset comorbidities in the OAMS population are still needed across vascular, musculoskeletal, cognitive, and psychiatric domains, as well as impact of hormonal fluctuations later in life for both male and female patients.

### Vascular disease and vascular risk factors

There is an increased risk of ischemic heart disease, congestive heart failure, stroke, and peripheral vascular disease in the MS population compared with overall population [81]. The cause of this increase is not clear—it may be driven by smoking, obesity, low physical activity, or immune abnormalities related to the disease process itself. Conversely, diabetes, hypertension, heart disease, hyperlipidemia, and peripheral vascular disease, when present at any point in the lifespan, are associated with more rapid progression of disability in MS patients [82]. In a population of adults with MS diagnosed at mean age 38.2 (SD 9.5 years), time from diagnosis to ambulatory assistance was median 18.8 years in patients without and 12.8 years in patients with vascular comorbidities. Moreover, synergistic impact of comorbidities has been noted. Depression was associated with increased risk of vascular disease in people with MS, which may further compound risk of increased disability [83]. While further research is required to determine impact of treatment of vascular comorbidities on MS-related disability accrual, recent studies are encouraging. Higher cardiovascular risk as measured by the Framingham risk score is associated with risk of relapse, disability and DMT escalation over a 5-year follow-up [78]. Treatment with statins has been associated with lower all-cause mortality rates, as well as cerebrovascular and macrovascular disease, in people with MS compared with people with MS not taking lipid-lower medications [84].

### Musculoskeletal conditions

Musculoskeletal comorbidities may uniquely predict physical ability in some studies. A study of patients with MS and musculoskeletal comorbidity showed a greater decline in physical function over 40 months compared to patients with MS without musculoskeletal comorbidity, controlling for age and MS disease severity [85]. Those with comorbidity were older (mean age 39.3 years, SD 9.6) versus those without (mean age 35.9 years, SD 9.5), though the impact specifically in older patients was not well examined, nor were specific musculoskeletal diagnoses provided.

### Comorbidities causing cognitive impairment

Cognitive impairment is common in MS. It is more prevalent in adults with MS than in age-matched peers, and 77.4% of OAMS over the age of 55 years have impairment in more than two cognitive domains [86]. It can occur at any point in the disease and occurs across MS subtypes, including clinically isolated syndrome, relapsing, and progressive forms [87]. Its prevalence is highest in progressive forms of MS, though age and physical disability may mediate this increased risk rather than the disease subtype itself [87, 88]. Specific domains of cognitive dysfunction and their severity vary between patients, but affected domains may include recent memory, sustained attention, verbal fluency, conceptual reasoning, and visuospatial perception [87]. Risk of certain deficits may change with age, as older and middle-aged adults over 40 years of age demonstrate worse visuospatial learning and memory than young adults, while adults over 60 years of age display worse cognitive processing speed than younger and middle-aged adults [89]. Age of MS onset may impact trajectory of cognitive impairment, as patients with LOMS have similar levels of physical disability to patients with adult onset MS but worse cognitive disability, particularly in visual memory, auditory working memory, and memory, even after controlling for disease duration and comorbidities [90].

Additionally, as patients age, medical comorbidities may increase the risk of cognitive concerns. In a cohort comprised partially of OAMS (mean age 51.2 years), vascular comorbidity was associated with lower cognitive function in OAMS, mediated by changes in brain imaging [91]. Moreover, additional comorbid dementia syndromes (such as vascular dementia or Alzheimer’s disease [AD]) may emerge. In ICD code-based population studies, the risk of vascular dementia was 3.75-fold higher in patients with MS compared with matched controls [92]. Further studies investigating AD and other primary dementia syndromes in patients with MS are needed, though recent studies do emphasize an increased risk of AD in patients with MS [92, 93]. However, it remains unclear whether risk factors for dementia in the non-MS patients, such as ApoE4 alleles, confer similar or higher levels of risk for AD in patients with MS or impact MS disease course. In some studies, ApoE4 has been a prognosticator of a globally more aggressive MS course, including physical disability progression, though not necessarily associated with an increased risk of cognitive dysfunction [94–96]. Differentiation of MS-related cognitive changes from other causes is important, and screening for other common causes, including metabolic, infectious, and neurodegenerative conditions is warranted when new-onset cognitive complaints are noted.

### Psychiatric comorbidities

At present, evidence-based guidelines do not consider age when screening for or treating psychiatric comorbidities in patients with MS [97]. However, OAMS may be especially at risk for undertreatment beginning in their mid-forties [97]. Depression and anxiety are the most common psychiatric comorbidities, with over 20% of patients affected and lifetime prevalence of 30–40%, depending on the measure used [81, 98–101]. Depression can occur at any age. When depression symptom scores of patients with MS over age 65 were compared with matched sample of young adults, OAMS reported fewer depressive symptoms than younger adults with MS [102]. However, MS-related helplessness was significantly higher in older compared to younger adults. Notably, in a cohort of patients with mean age of MS onset in their early 30 s, the prevalence of antidepressant use increased from 24.1% in 18–29-year-olds to 32.3% in patients over 60 years [103]. Prevalence of moderate to severe depression was 9.3% in 18- to 29-year-olds and fell to 7.8% in patients over 60.

Of note, psychiatric comorbidities heavily impact cognitive function in patients with MS. When present, severe depression causes changes in working memory, executive function, and reduced information processing speed (similar to those with severe depression without MS). This is particularly true for capacity-demanding tasks, with longer time and increased errors on planning tasks in patients with MS with depression [104, 105].

Anxiety tends to occur in younger patients and has been associated with shorter disease duration, though with notable overlap with depression [106]. Comorbid depression and anxiety lead to consistently low levels of psychological well-being and quality of life [107]. Anxiety has also been shown to impact processing speed [80]. Robust data on prevalence in adult onset MS are lacking.

Practical strategies for addressing psychiatric comorbidities include identifying anxiety, addressing substance use, and promoting increased social support to mitigate the impact of depression and anxiety. Transdiagnostic psychiatric treatment is appropriate when comorbidities are present [106].

### Menopause

Approximately 30% of the MS population comprises women who are peri- or menopausal [108]. Age of menopause has not been found to differ in women with MS compared with the general population, with a mean age of around 51 years [108–110]. Symptoms of menopause can overlap those of MS, including changes in cognition, mood, bladder function, and sleep [109]. Some studies suggest reduced relapse rate and increased disability progression post-menopause, though systematic review of 28 studies generally did not find difference in disability accumulation pre- and post-menopause [111–113].

Little is known about the impact of hormonal replacement therapy (HRT) on MS disease course [108]. In the Comprehensive Longitudinal Investigation of MS at the Brigham and Women's Hospital (CLIMB) study, 16.2% of women had used estrogen HRT, either alone or in combination with progesterone within 5 years of menopause [110]. Use of HRT may be beneficial with regard to quality of life [114]. Smaller studies have shown tolerability of some hormonal therapies, but no large studies have examined risks/benefits in-depth for post-menopausal women with MS [115–117]. There are no studies at this time that guide DMT selection specifically with respect to pre- or post-menopausal state.

### Wellness and preservation of neurological reserve in older populations

Wellness and preventative care are an important aspect of management in OAMS. These interventions are valuable at all ages, but are particularly relevant in older age.

Smoking is associated with accelerated brain atrophy and disability worsening in patients with RRMS and worsening long-term disability as measured on the Timed-25-Foot Walk and Paced Auditory Serial Addition Test [118]. Following smoking cessation, the rate of motor deterioration slows and eventually matches that in those who never smoked [119]. These observations emphasize the value of smoking cessation.

Exercise is another component to maximal health and has been proposed to be a disease-modifying intervention [120]. In a randomized cross-over trial of progressive resistance training in 35 adults (mean age 43 years), increased cortical thickness and radiographic changes after 24 weeks were noted [121]. While the precise mechanism is not known, physical training interventions in persons with MS improve vascular risk factors and may be considered as a therapeutic strategy for managing vascular comorbidities [122].

Social determinants of health are also critical and may play an increased role with aging. Economic components, including fixed income, race, isolation, transport, living environment, and lack of employment all need to be considered in the context of care for older MS patients [123]. Together, these may contribute to up to 55% of health outcomes.

## Conclusion and future directions

**Fig. 2:**
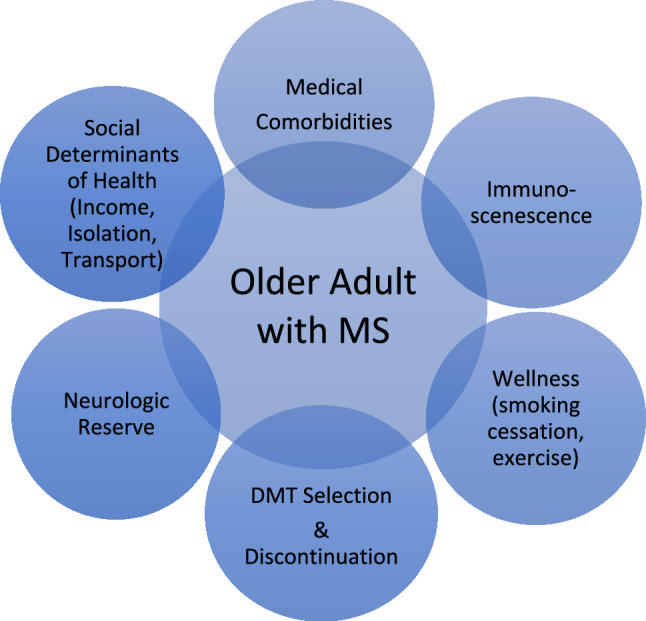
Special considerations in the older adult with MS

In this review, we highlight key considerations for comprehensive care of people with MS as they age (Fig. 2). Despite the increasing number of OAMS, research in diagnosis and management of this MS population is limited. Gaps in knowledge persist surrounding LOMS diagnosis, OAMS disease behavior, utility of DMTs, and management of comorbidities. Research that includes the impact of comorbidities on disease progression specifically in older patients would be useful, and ideally studies would report age of comorbidity onset, severity, and treatment status to clarify this relationship further. Clinical trials particularly have underrepresentation of the aging MS population. Inclusion of OAMS in future clinical trials should be a priority. Furthermore, efforts into continued MS epidemiologic studies remain paramount in order to better understand factors that influence MS development, pathophysiology, and disease course over time.

## Data Availability

Data availability statement is not applicable as this review article is based exclusively on published work.
